# Severe Crowding Associated with Lower Canine Premature Resorption: Interceptive Treatment with Clear Aligners—A Pilot Study

**DOI:** 10.3390/children11040451

**Published:** 2024-04-08

**Authors:** Francesca Gazzani, Chiara Pavoni, Saveria Loberto, Silvia Caruso, Paola Cozza

**Affiliations:** 1 Department of Clinical Sciences and Translational Medicine, University of Rome ‘Tor Vergata’, 00133 Rome, Italy; francescagazzani@hotmail.it (F.G.); saveria.loberto@gmail.com (S.L.); 2 Department of Faculty of Medicine and Surgery, UniCamillus International Medical University, 00131 Rome, Italy; chiara.pavoni@unicamillus.org; 3 Department of Life, Health and Environmental Sciences, University of L’Aquila, 67100 L’Aquila, Italy; silvia.caruso@univaq.it

**Keywords:** early mixed dentition, lower incisors crowding, arch development, clear aligner treatment

## Abstract

Background: Early mixed dentition represents a critical phase since crowding conditions can occur. The interceptive resolution of dental crowding allows favorable arch and occlusal development. The aim of the present investigation was to evaluate dentoalveolar changes of clear aligner treatment planned to manage lower incisor crowding, loss of arch length, and midline deviation in early mixed dentition. Methods: A total of 13 patients (7 females, 6 males, 9.4 ± 1.2 age) treated with clear aligners were selected. Arch dimensions and incisor inclinations were evaluated before (T0) and at the end of interceptive treatment (T1). A paired *t*-test was chosen to compare T1–T0 changes. The level of significance was set at 5%. Results: The greatest significant increase in mandibular width was observed at the level of the first deciduous molars (+2.44 ± 1.4 mm), followed by the second permanent molars (+2.16 ± 1.4 mm). Lower arch length and arch depth showed a statistically relevant increase (2 ± 0.6 mm and 4.5 ± 1.6 mm, respectively). The mean lower dental midline changes were statistically significant (1.42 ± 0.73 mm). Conclusions: Early treatment with clear aligners, including the combination of transversal arch development, maintenance of leeway space, and guidance of eruption, represents a valid treatment strategy in early mixed dentition to manage arch crowding and occlusion development.

## 1. Introduction

The early mixed dentition stage in growing patients starts approximately at the age of 6 with the eruption of the permanent incisors, first molars, and the establishment of the occlusion. This moment represents a critical occlusal development phase since the eruption of permanent incisors requires space in both arches, especially in the mandibular one. For a perfect alignment, an average space deficit of 1.6 mm is observed during lower incisor eruption [1,2]. In the transitional dentition, a slight crowding up to 2–3 mm at the level of lower incisors can be considered normal, and it may be solved spontaneously during later stages of occlusal development [2]. On the other hand, crowding conditions are considered more severe when incisor eruption is associated with the early loss of primary teeth. Tooth loss can be considered premature when it occurs at least one year before the normal exfoliation period as a consequence of an atypical inflammatory process [1]. Several causes were identified as determining factors, such as trauma, dental caries, and premature extraction. However, tooth size discrepancies between primary and permanent teeth represent one of the main reasons related to this phenomenon [1,2,3,4,5,6,7,8,9]. Dental crowding in the mixed dentition represents one of the most frequent malocclusions. Primary lower canines and upper lateral incisors are the most affected teeth, followed by upper canines, lower lateral incisors, and second molars [1,2,3,4,5,6,7,8,9]. When the eruption of permanent mandibular incisors determines the premature resorption and exfoliation of the adjacent deciduous canine [8], the Atypical Premature Root Canine Resorption (APRCP) can be observed. APRCP is stated when the loss of a deciduous canine occurs in the early mixed dentition phase, long before its physiological time of exfoliation. This significant clinical manifestation occurs as a clinical sign of primary crowding, which often induces a reduction of the arch length perimeter, the mesial migration of posterior arch segments, and a worsening of the deep bite [1,3]. Moreover, when premature loss of the deciduous canine is unilateral, a displacement of the midline towards the side of the loss and sagittal asymmetry of the arch perimeter can be observed [10,11,12,13]. Lower incisor crowding and the need for early treatment have long been a topic of debate in the literature [2,3,4,10,11,12,13]. Some authors concluded that the premature loss of anterior teeth does not compromise arch perimeter and length as much as early exfoliation of posterior teeth does [1,13]. On the other hand, others support the importance of an early approach for a timely recovery of midline coordination, sagittal lower arch symmetry, and management of dentition spaces [8,12,13,14,15,16,17,18,19]. Interceptive orthodontic treatment in early mixed dentition has the objective of allowing a more favorable arch development and a consequent balanced occlusion. From a biomechanical and treatment planning point of view, dentoalveolar crowding requires the combination of several strategies, including transversal expansion, recovery of proper crown inclination, space management, and possible planned extractions. Transversal expansion strategies in the mandibular arch are limited to the dentoalveolar components because of the lack of a midline suture. For this reason, space management through timely extraction and guidance of eruption represents the main clinical strategy indicated. Other strategies used for space-gaining purposes in the anterior region consist of labiolingual inclination of lower incisors and stripping procedures. An increase in vestibular inclination of lower incisors can compromise the maintenance of the anterior limit of the dentition. Thus, it should be planned only in the presence of reduced values of IMPA considering the periodontal features and the skeletal vertical components [10]. Stripping procedures are not recommended for permanent teeth during the mixed dentition stages because of the available space recovery potentialities, such as the leeway space [10]. Some authors [20] suggested the reduction of the primary canine when a lower incisor crowding of 2–4 mm is present. This procedure provides additional space to enhance the position of the permanent incisors but requires intact roots of deciduous canines without resorption changes due to the erupting lateral incisors. As for the extraction protocol, in the case of unilateral canine exfoliation, the extraction of the corresponding tooth on the other side has been suggested [4,10,14]. Giannelly concluded that the combination of opposing canine extraction and placement of a lingual arch can help control midline coordination, symmetry, and arch length [10]. One of the main advantages of clear aligners in the early mixed dentition stage is represented by the possibility of planning and obtaining simultaneous dental changes while maintaining the arch perimeter and controlling lower incisor inclination [16]. The extension of clear aligner treatment for the mixed dentition provided efficient strategies for Phase I therapeutic needs [21]. Hence, the aim of the present study was to evaluate dentoalveolar changes obtained by clear aligner treatment planned to manage lower incisor crowding, loss of arch length, and midline deviation in early mixed dentition.

## 2. Materials and Methods

The present pilot study was designed as a prospective observation study. The research project was approved by the Ethical Committee of the Hospital of Rome “Tor Vergata” (protocol number 163.20, 23 July 2020). Written informed consent was obtained from all patients’ parents. A total of 13 patients (7 females, 6 males, 9.4 ± 1.2 years) who sought an orthodontic treatment were enrolled in the study group. All subjects showed the following inclusion criteria: European ancestry, early mixed dentition stage with fully erupted first permanent molars, dentoalveolar transverse discrepancy up to 5 mm, mesial step or a flush terminal plane molar relationship, a normal inclination of lower incisors (IMPA angle 89–90°), anterior severe mandibular crowding between 5 and 8 mm, dental lower midline deviation > 2 mm, premature exfoliation of one or both deciduous canines, good compliance with aligners, and pre- and post-treatment intraoral scan data and lateral cephalograms. Patients with one of the following exclusion criteria were not included in the study: multiple and/or advanced caries, tooth agenesis, supernumerary teeth, cleft lip and/or palate, oral habits, previous orthodontic therapy, and use of other auxiliary appliances.

### 2.1. Treatment Protocol

All patients underwent treatment with clear aligners Invisalign First System^®^ (Align Technology, Santa Clara, CA, USA). No auxiliaries other than attachments nor interproximal enamel reduction (IPR) were required. The treatment plan and ClinCheck^®^ (Align Technology, Santa Clara, CA, USA) for each patient were scheduled with the same staging to obtain crowding resolution, dental midline correction, and recovery of sagittal symmetry. The standardized APRCP treatment protocol is composed of transverse expansion in both arches and 8 sequential movements digitally planned in the lower arch. Sequential staging “molars move first” expansion pattern, followed by simultaneous expansion of all posterior deciduous teeth and canines, was planned in the upper and lower arch [15,22]. “Molars move first” expansion included a mesial-out rotation of maxillary first molars (2 degrees for each aligner) following the Rickett’s line [15]. Concerning the lower arch, the following staging was planned to manage the APRCP: (1) extraction of the opposite, deciduous canine in case of asymmetric exfoliation of only one primary canine; (2) distorotation of mandibular first molars and posterior limit definition; (3) alignment and crown torque control of lower incisors; (4) labiolingual inclination of lower incisors, arch length increase, and root torque control; (5) check of anterior limit of the dentition by means of a digital overlapping tool; (6) active eruption compensator in correspondence of the lower permanent canine to gain enough space for its eruption; (7) recovery of midline shift; (8) expansion of lateral segments. All patients were instructed to wear the aligners full-time except during meals and tooth-brushing activities. The aligners were changed every 7 days during the entire treatment. For every 4 stages corresponding to 4 aligners, the clinician checked the aligner fit, the movements’ correspondence with the virtual treatment plan, and the attachments’ positioning. In case of loss of fit due to tooth exfoliation or eruption, the original prescription was adopted to continue the first approved treatment plan on the ClinCheck Pro^®^ software. Patients were aware to be part of a research study at the delivery appointment. A single investigator performed a follow-up interview with each patient to evaluate the level of compliance. A three-point (poor, moderate, good) Likert-type scale was adopted to evaluate Patient’s collaboration [15,22] according to the following criteria: low compliance when the patient wore the aligners for less than 16 h per day; moderate from 16 to 20 h per day; good when the aligners were worn full time as indicated. The mean number of aligners was 45 for both the maxillary and mandibular arch. Lateral cephalograms and digital models were collected for each patient before starting the treatment (T0) and at the end of the first phase of the interceptive treatment (T1). The average time between T0 and T1 was equal to 12.5 months.

### 2.2. Measurements

**Figure 1:**
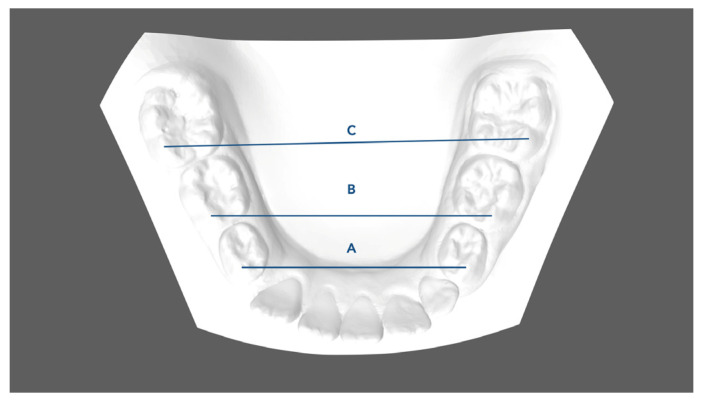
Dental arch width measurements: A. Mandibular first inter-deciduous molar width (IV–IV); B. Mandibular second inter-deciduous molar width (V–V); C. Mandibular inter-molar width (6–6): linear distance between the mesio-buccal cusp tips of the right and left mandibular first molars.

**Figure 2:**
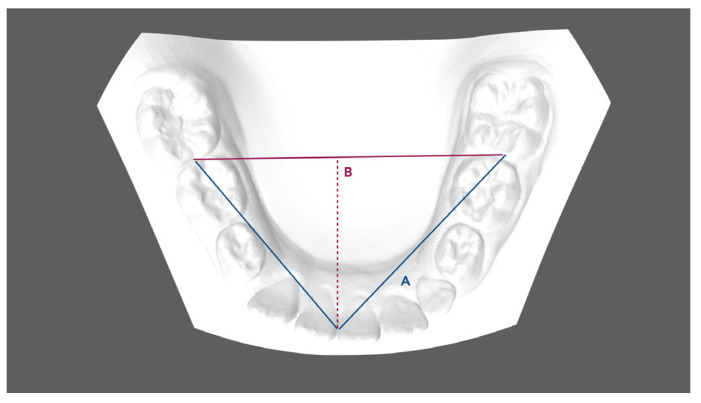
Dental arch width measurements: A. Arch length, B. Arch depth.

**Figure 3:**
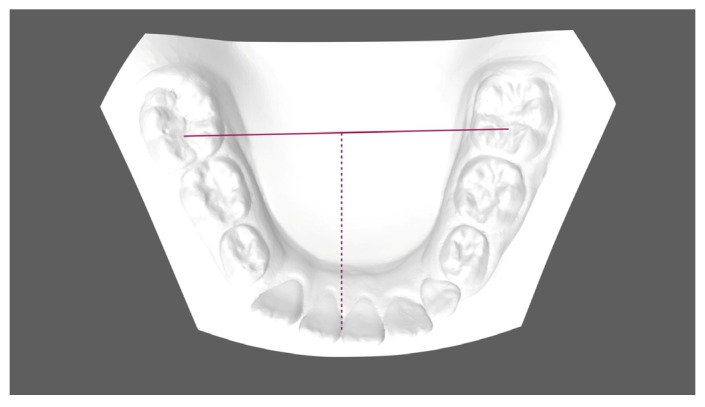
Mandibular midline position.

Pre- (T0) and post-treatment (T1) digital scans were obtained for all subjects through the intraoral scanner iTero^®^ Orthodontic ver. 5.2.1.290 (Align Technology Inc., Santa Clara, CA, USA). One investigator (FG) performed all the measurements. Digital models (.stl models) were created and then uploaded to Viewbox 4 software (dHAL software, Kifissia, Greece) to perform the digitization, and the measurements required on the digital casts were created. The following linear values were measured for each T0 and T1 model to evaluate any changes in terms of arch dimension and shape (Figure 1, Figure 2 and Figure 3):

- Mandibular first deciduous inter-molar width (IV–IV): linear distance evaluated between the mesio-buccal cusp tips of the right and left mandibular first deciduous molars;
- Mandibular second deciduous inter-molar width (V–V): linear distance evaluated between the mesio-buccal cusp tips of the right and left mandibular second deciduous molars;
- Mandibular inter-molar width (6–6): linear distance evaluated between the mesio-buccal cusp tips of the right and left mandibular first permanent molars;
- Arch length: sum of the right and left distances traced from mesial anatomic contact points of the first permanent molars to the contact point of the central incisors;
- Arch depth: perpendicular length from the midpoint drawn between the mandibular central incisors to the line drawn between the mesial anatomic contact points of the first molars;
- Midline discrepancy: difference between the initial and final position of the lower midline. The mandibular midline position was determined by measuring the distance between the contact point of the central incisors and the first molar central fossa line.

Incisor inclination changes observed in the interval T0–T1 were evaluated on lateral cephalograms by adopting the following:

- IMPA (°): angle between the long axis of the lower incisor and the mandibular plane (Go-Me).
- L1-APg (mm): linear distance between the lower crown tip and the A-Pg line.

### 2.3. Statistical Analysis

The sample size was calculated according to the method proposed by Whitehead et al. [23,24]. For the standardized effect size of 1, a clinically relevant change of 0.35 mm with a combined SD of 1.10 for the primary variable mandibular inter-molar width, a sample size of 13 subjects was required for an error rate of 5% and a power of 80%. The measurements were analyzed with the interclass correlation coefficient (ICC). Sample normality was tested using the Shapiro–Wilk test. In the presence of normally distributed data, a paired t-test was selected to compare T0–T1 changes. To determine the reliability of the method, 10 digital casts randomly chosen were measured by the same operator on two separate occasions at least 1 month apart. A paired *t*-test was used to evaluate the systematic error. The level of significance was set at 5%. SPSS (Statistical Package for the Social Sciences), version 18.0 (IBM Corp, Chicago, IL, USA), was the selected software to analyze the data.

## 3. Results

The recruitment started in November 2021, and the observation period ended in March 2023. Compliance evaluation of the treated patients (use of aligners) highlighted a good/moderate level of cooperation in all of them. Table 1 shows the mean values (±SD) and the differences between the T0 and T1 arch dimensions and midline changes. 

**Figure 4:**
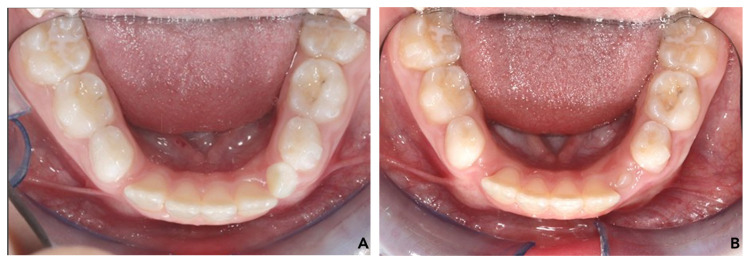
Dental arch dimensions and midline deviation. (**A**) Before treatment (T0); (**B**) at the end of interceptive treatment (T1).

Statistically significant differences were observed between pre- and post-treatment outcomes, except for the second deciduous inter-molar measurements (Figure 4). The greatest significant increase in mandibular width was observed at the level of the first deciduous molars (+2.44 ± 1.4 mm; *p* < 0.001), followed by the second permanent molars (+2.16 ± 1.4 mm; *p* < 0.001). A lower increase was observed at the first deciduous molars, although not significant (+1.66 ± 1.5 mm; *p* < 0.01). Regarding lower arch length and depth dimensions, all measurements revealed significant T0–T1 changes. Lower arch length and arch depth showed a statistically relevant increase (2 mm ± 0.6 mm and 4.5 ± 1.6 mm, respectively). The mean lower dental midline changes were statistically significant in the T0–T1 interval (1.42 ± 0.73 mm). When analyzing the inclination of lower incisors, an increased proclination was observed despite not being statistically significant (IMPA, 1.4° ± 1.36°; L1-APg, 1.6 mm ± 0.5 mm).

## 4. Discussion

Crowding and space issues are the most prevalent components of malocclusions in growing patients. Understanding the etiology of crowding can help clinicians identify potential needs for early intervention. Some authors have supported the spontaneous resolution of mild crowding [2,3]. Van der Linden stated that crowded and ectopic lower incisors can gradually find a better repositioning during eruption [25]. According to Proffit et al. [2], mildly crowded lower incisors easily find extra space for spontaneous resolution from jaw growth processes such as width increase of dental arches, labial positioning of permanent incisors relative to primary teeth, and slight back repositioning of mandibular canines during permanent incisor eruption. Nevertheless, in more severe cases, the eruption of lateral incisors can lead to a premature loss of primary canines, causing the deviation of the dental midline, migration of posterior segments, and reduction of the arch perimeter [16,21,26,27,28,29,30,31,32,33]. Arch length discrepancies derived from severe crowding conditions can determine more severe malocclusion, ectopic eruption, rotation, impaction of permanent teeth, worsening of overbite and overjet, and an unfavorable molar relationship. In these cases, an interceptive approach and proper management of space in mixed dentition can promote permanent tooth eruption and prevent loss of arch length [26]. According to many authors, a well-timed intervention can reduce severe incisor crowding, increase the long-term stability of their position, decrease the phenomena of ectopic eruption or impaction, and improve gingival and dental health [27,28]. Several treatment strategies have been introduced over the years for the early management of crowding in early mixed dentition. Some authors [31,32] suggested the use of space maintainers or lingual arch to contrast the early loss of primary teeth. As confirmed by Giannelly [10], in mixed dentition, arch length preservation, by means of the maintenance of leeway space, can provide adequate space to resolve lower incisor crowding. For length preservation treatment, Brennan et al. [33] proposed the use of a passive lingual arch that, along with developmental changes in transitional dentition, would provide up to 4–5 mm of crowding correction. Lingual arches are designed to passively preserve arch length by avoiding mesial migration of posterior segments and by preserving the leeway space for developmental modification in the transitional dentition. However, adverse effects associated with these passive devices have been described in the literature in terms of oral hygiene conditions, caries, interference with eruption processes, undesirable tooth movement, and soft tissue injuries [30,31]. More recently, the application of clear aligners has resulted in particularly advantageous results in mixed dentition in terms of arch length recovery, beneficial use of the leeway space, and arch development. The main advantage is represented by the possibility of planning movements in all three planes of space and simultaneously managing eruption processes. To the best of our knowledge, a limited number of studies have analyzed the effects and effectiveness of clear aligners in mixed dentition [15,21,22,24,30,31,32,33,34,35,36]. Thus, the aim of the herein investigation was to evaluate arch modification changes obtained by Invisalign First^®^ clear aligners in the resolution of crowding in mixed dentition. The APRCR clinical protocol was designed to manage the developing arch form, as well as recover the loss of space, solve anterior crowding, and manage the anterior limit of dentition throughout a controlled labio-inclination of lower incisors. An increase in transverse dimensions occurred in all analyzed patients. The extent of the obtained expansion was greater at the level of the first permanent molars and second deciduous molars (+2.44 ± 1.4 mm; +2.16 ± 1.4 mm, respectively). Indeed, arch development on the transversal plane shows beneficial effects on the transition from primary to secondary dentition by developing extra arch length and space in labial arch segments [33]. Germane et al. [31] concluded that a 1 mm increase in inter-molar width resulted in a 0.27 mm increase in the arch perimeter. Significant changes were observed in terms of length and depth dimensions (2 mm ± 0.6 mm and 4.5 ± 1.6 mm, respectively) (Figure 4). The recovery of arch length was supported by the combination of transversal arch development, proclination of permanent incisors, and maintenance of leeway space. The slight and not statistically relevant modifications of lower incisors (IMPA, +1.4° ± 1.36°; *p* > 0.5; L1-APg, +1.6 mm ± 0.5 mm; *p* > 0.5) showed a good control of their inclination allowing alignment and sagittal development. Moreover, the possibility of integrating additional features, such as the active eruption compensator into the aligners, advantageously manages the interproximal leeway space and recovers the loss of anchorage secondary to the mesial migration of posterior segments [30,31]. Lip bumper therapy represents another treatment strategy used over time to gain arch length [37,38]. The claimed effect is a forward bodily incisor movement and flaring of the lower incisors, which can easily compromise the anterior limit of dentition. Thus, the use of clear aligners can be considered an additional treatment strategy for severe crowding cases to obtain transversal dentoalveolar expansion, to align the lower incisors by controlling labio-inclination movements, and the recovery of non-coincident midlines in mixed dentition. Limitations of the herein investigation regarded its preliminary purpose, the absence of a control group, and the small sample size. Further research with a larger sample and a control group for a comparative evaluation would be necessary to strengthen the obtained results and to better understand the management of early mixed dentition with clear aligners.

## 5. Conclusions

- Atypical Premature Root Canine Resorption (APRCP) is a clinical sign of primary crowding, which requires accurate space management during early mixed dentition.
- Early treatment with clear aligners represents a valid treatment strategy in early mixed dentition to manage arch and occlusion development.
- The combination of transversal arch development, control of permanent incisor inclination, maintenance of leeway space, and guidance of eruption allows the early recovery of anterior dental crowing and arch length discrepancies.
- Further studies with larger sample sizes and longer observational periods are needed to strengthen the described dentoalveolar changes.

## Figures and Tables

**Table 1:** Descriptive statistics and statistical comparisons of T1–T0 changes (paired *t*-test).

**Variables**	**Pre-Treatment (T0)**		**Post-Treatment (T1)**		**T1–T0**		
**Arch Dimension Measurements (mm)**	**Mean**	**SD**	**Mean**	**SD**	**Diff**	**SD**	***p* Value**
First inter-deciduous molar width (IV–IV)	38.2	1.4	39.8	1.8	1.6	1.5	0.01
Second inter-deciduous molar width (V–V)	29.6	0.9	31.7	2.2	2.1	1.4	0.001 *
Mandibular inter-molar width (6–6)	43.7	2.2	46.1	2.1	2.4	1.4	0.001 *
Arch Length	63.4	3.0	65.7	0.9	2.3	0.6	0.001 *
Arch Depth	27.5	2.1	32	2.2	4.5	1.6	0.05 *
Midline Discrepancy	2.2	0.9	0.3	1.8	−1.9	0.7	0.01 *
**Incisors’ Inclination Changes**	**Pre-Treatment (T0)**		**Post-Treatment (T1)**		**T1–T0**		
IMPA (°)	90.6	2.5	91.8	1.2	1.2	1.3	0.5
L1-APg (mm)	2.3	1.9	3.9	1.9	1.6	0.5	0.5

Mm, millimeter; SD, standard deviation; ° degree. * *p* < 0.05.

## Data Availability

The original contributions presented in the study are included in the article, further inquiries can be directed to the corresponding author.
